# Detecting native and bioprosthetic aortic valve disease using ^18^F-sodium fluoride: Clinical implications

**DOI:** 10.1007/s12350-020-02411-x

**Published:** 2020-11-11

**Authors:** Alexander J. Fletcher, Marc R. Dweck

**Affiliations:** British Heart Foundation Centre for Cardiovascular Science, University of Edinburgh, Chancellor’s Building, 49 Little France, Edinburgh, EH16 4TJ UK

**Keywords:** Aortic stenosis, Molecular imaging, Positron emission tomography, Surgical valve replacement

## Abstract

Calcific aortic valve disease is the most common valvular disease and confers significant morbidity and mortality. There are currently no medical therapies that successfully halt or reverse the disease progression, making surgical replacement the only treatment currently available. The majority of patients will receive a bioprosthetic valve, which themselves are prone to degeneration and may also need replaced, adding to the already substantial healthcare burden of aortic stenosis. Echocardiography and computed tomography can identify late-stage manifestations of the disease process affecting native and bioprosthetic aortic valves but cannot detect or quantify early molecular changes. ^18^F-fluoride positron emission tomography, on the other hand, can non-invasively and sensitively assess disease activity in the valves. The current review outlines the pivotal role this novel molecular imaging technique has played in improving our understanding of native and bioprosthetic aortic valve disease, as well as providing insights into its feasibility as an important future research and clinical tool.

**Electronic supplementary material:**

The online version of this article (10.1007/s12350-020-02411-x) contains supplementary material, which is available to authorized users.

## Introduction

Aortic stenosis affects 1-2% of the general population >65 years old, conferring with it increased mortality.^1^,^2^ Current international guidance recommends that the aortic valve should be replacement in those with severe, symptomatic aortic stenosis; a procedure which carries morbidity and significant cost.^1^,^3^–^5^ Replacement with a bioprosthetic valve is recommended in those over 65-70 years old, with mechanical valves preferred in the minority of younger patients.^6^ Bioprosthetic valves are beneficial in that they do not require the life-long anticoagulation that comes with mechanical valve implantation; however, they are prone to degradation over a relatively short time-span of ~15 years.^6^ Medical therapies that prevent progression of native or bioprosthetic aortic valve disease would represent a significant step in the management of these patient groups, but have so far remained elusive. To develop successful targeted medical therapy, identification of the mechanisms driving the disease process is crucial. Conventional imaging with computed tomography (CT) or transthoracic echocardiography (TTE) can identify the structural and haemodynamic manifestations of valvular disease; however, they cannot provide information about the molecular processes underpinning valve disease. Molecular imaging, on the other hand, can identify and quantify disease activity non-invasively and has an emerging role in evaluating the efficacy of medical therapies in randomized controlled trials as well a possible clinical utility in the early detection of aggressive disease.^7^ The current review outlines the pivotal role molecular imaging has played in our understanding of disease mechanisms, as well as providing insights into its feasibility as an important future research and clinical tool in the setting of native and bioprosthetic aortic valve disease.

## Disease of the Native Aortic Valve

The aortic valve is located at the junction between the left ventricle and aorta and functions to prevent backflow of blood into the left ventricle during diastole while allowing unimpaired systolic ejection. Anatomically, the aortic valve consists of three leaflets that are anchored to a crown-like anulus. During diastole, the high aortic pressures force the leaflets to coapt, closing the valve and forming three blood-filled sinuses; two of which contain coronary arteries and facilitate blood flow. The aortic leaflets are highly specialized structures that must be compliant enough to open without resistance to blood flow, while being strong enough to withstand the repeated mechanical stresses applied throughout the cardiac cycle. In aortic stenosis, diseased valve leaflets become stiff and lose compliance, increasing the intraventricular pressure required to generate the same flow across the valve. The current model of native aortic valve pathology is thought to involve an initiation phase, where initial valvular injury leads to inflammation, immune activation and initial calcium deposition, followed by a propagation phase, involving a damaging cycle of increasing calcification activity.^8^ Increasing haemodynamic resistance across the valve can be identified with transthoracic echocardiography (TTE), while end-stage structural valve disease can be seen as valvular calcification identified on computed tomography (CT). Both TTE and CT calcium scoring have been integrated into international guidelines to determine when to perform aortic valve replacement.^3^,^4^ However, by the time haemodynamic changes or overt calcific disease are seen on TTE or CT, these disease processes are already well under way. The development of a successful medical therapy will depend on identifying and understanding the details of the active disease processes leading to these structural and haemodynamic changes.

## Calcification as the Driver of Disease in Native Aortic Valve Stenosis

Due to a strong overlap between risk factors for developing atherosclerosis and aortic stenosis, the lipid-lowering effect of statins was initially investigated as a means of slowing aortic stenosis progression. Frustratingly, multiple randomized controlled trials did not demonstrate a reduction in aortic valve velocity or calcium score progression over medium-term follow-up.^7^,^9^ While disappointing, the results also questioned the understood inflammatory mechanisms driving the process of aortic stenosis.

**Figure 1:**
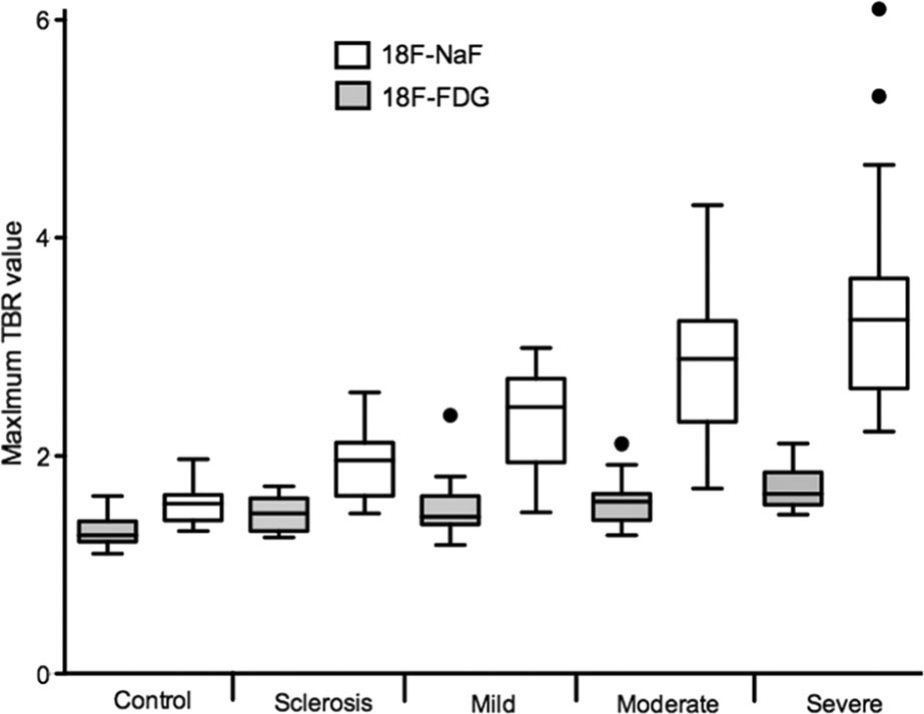
Uptake of 18F-fluorodeoxyglucose (^18^F-FDG) and ^18^F-sodium fluoride (^18^F-NaF) according to the severity of aortic stenosis. Box plots show the median and interquartile ranges of the tissue-to-background ratios (TBR) for ^18^F-NaF (white boxes) and ^18^F-FDG (gray boxes) with whiskers to 1.5 interquartile range. Figure reproduced with permission from Dweck et al.^10^

**Figure 2:**
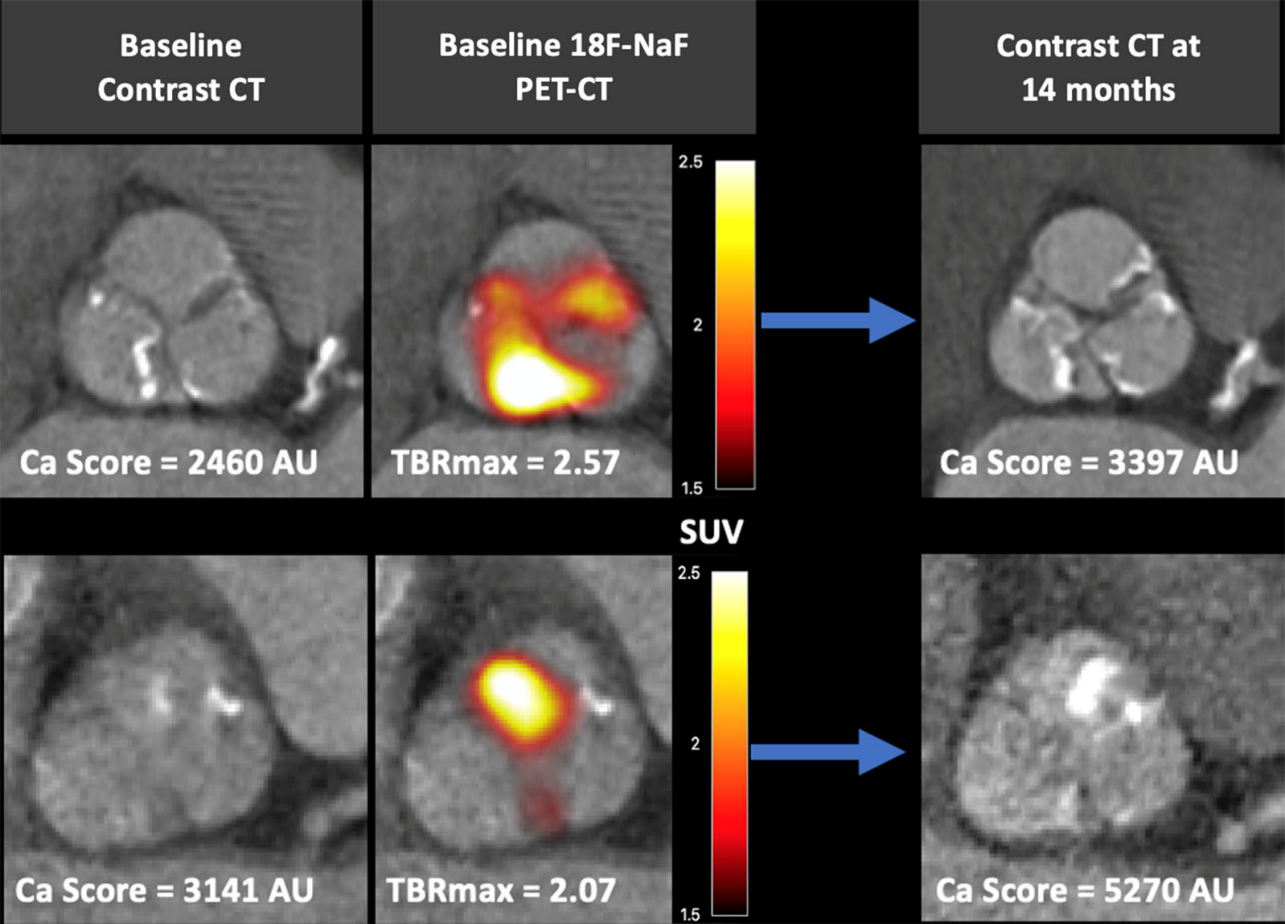
Two patients with progressive calcific aortic valve disease. (Left) Baseline computed tomography (CT) images. (Middle) Fused positron emission tomography (PET)-CT images showing increased ^18^F-fluoride valvular uptake (red/yellow areas). (Right) Repeat CT scans after 14 months with progressive aortic valve calcium score and new macroscopic calcium (white areas) in a similar distribution to that of baseline PET uptake. *18F-NaF PET-CT*, ^18^F-sodium fluoride; *PET*, positron emission tomography; *CT*, computed tomography; *Ca Score*, Calcium score in Agatston units; *TBRmax*, maximum tissue-to-background ratio

To explore the relative contributions of inflammatory and calcific processes to aortic stenosis *in vivo*, researchers performed hybrid positron emission tomography combined with computed tomography (PET/CT), comparing ^18^F-fluorodeoxygloucose, (^18^F-FGD), a non-specific marker of inflammation, and ^18^F-sodium fluoride (^18^F-NaF), which preferentially binds to developing microcalcification, in a cohort of patients with varying degrees of aortic stenosis. The results demonstrated that calcification activity dominated over inflammation in aortic stenosis, particularly in the latter stages of moderate or severe stenosis (Fig. 1).^10^ The reverse situation was observed in concomitant regions of atheroma, with inflammation predominating: an observation that perhaps explains the differential effects of statins in these two conditions.^11^ Importantly, the anatomical pattern of ^18^F-NaF uptake at baseline was different to the presence of baseline calcium on CT, confirming that these two modalities provide different information. However, this baseline ^18^F-NaF PET activity did predict where new regions of calcium on CT would develop after 1-2 years of follow-up (Fig. 2). Similar observations have been made in different cardiovascular conditions (e.g., mitral annular calcification and coronary atherosclerosis)^12^,^13^ supporting ^18^F-NaF PET as a marker of newly developing calcification beyond the resolution of CT and as a marker of disease activity in aortic stenosis. Overall in the population of 121 patients, baseline ^18^F-NaF closely predicted progression of aortic valve calcium score after 2 years (*R* = .80, *P* < .001) as well as clinical events (aortic valve replacement or death).^14^,^15^

## Accurately Measuring Aortic Valve ^18^F-Sodium Fluoride Uptake

**Figure 3:**
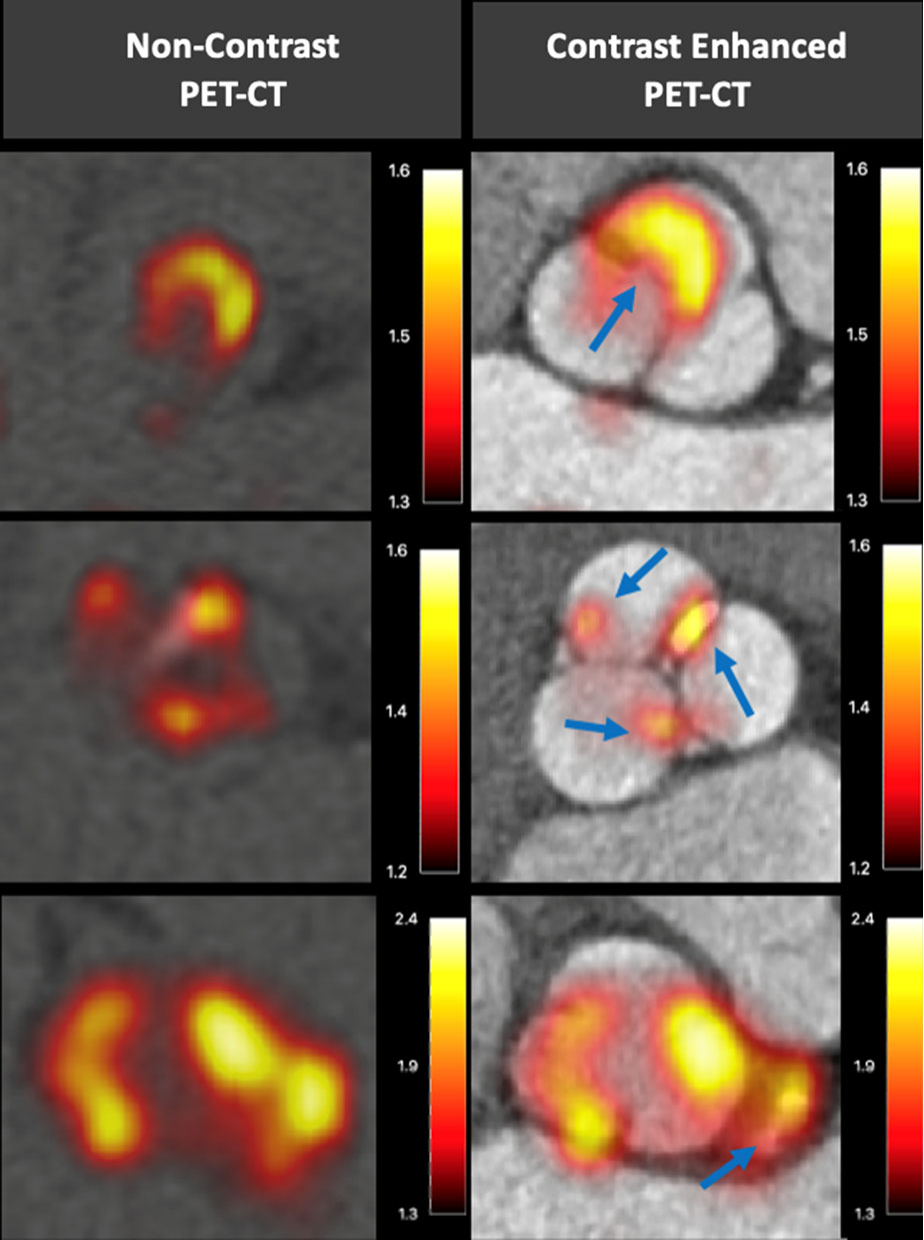
Improved localization of positron emission tomography (PET) signal within the aortic valve and its leaflets. Paired non-contrast PET-computed tomography (CT) scans (left) and contrast-enhanced PET-CT images (right). Images demonstrate the typical distribution of the tracer uptake within the valve at sites of increased mechanical stress, that is, at the leaflet tips (top, blue arrow) and at the commissures (middle, blue arrows). Contrast enhancement also aids identification of valvular and peri-valvular uptake, for example in the coronary arteries (bottom, blue arrow). Scale bars represent standardized uptake values (SUV). *PET*, positron emission tomography; *CT*, computed tomography

**Figure 4:**
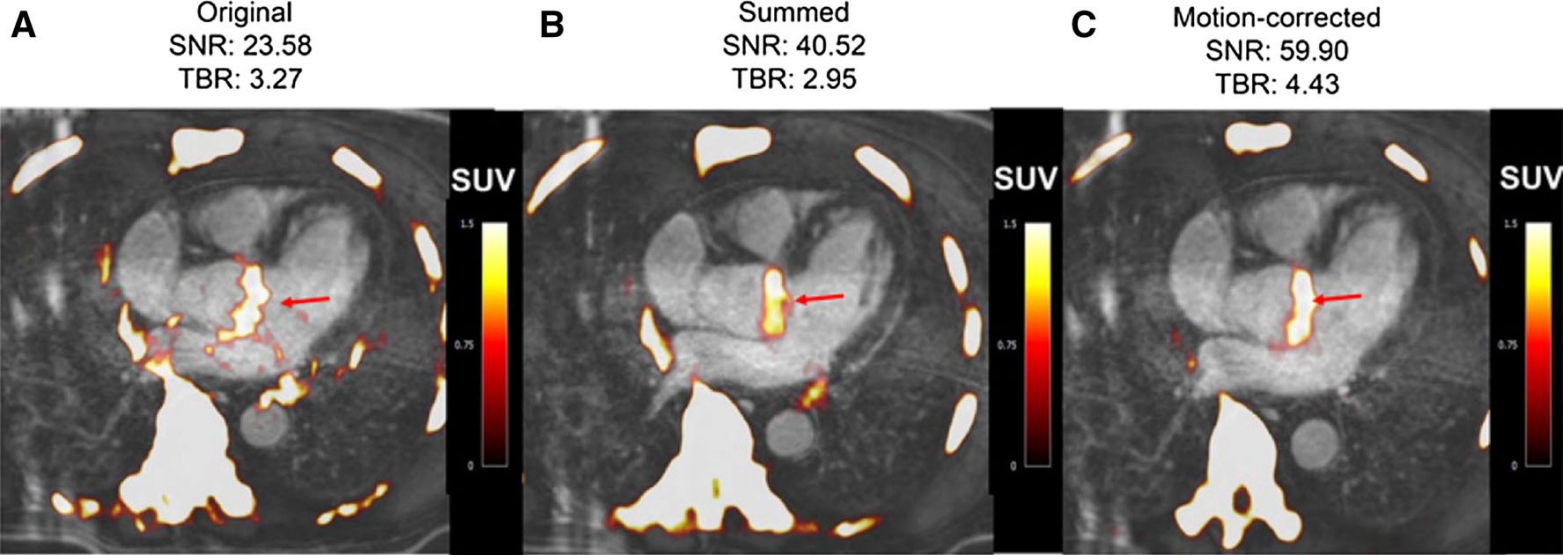
Fused ^18^F-fluoride PET/contrast-enhanced MR angiograms of aortic valve of 60-year old man with aortic stenosis. Shown are original diastolic gate (**A**), summed image (**B**), and motion-corrected image (**C**) with focal ^18^F-fluoride uptake (arrows). This research was originally published in JNM. Doris et al.^19^ © SNMMI

While the early studies described above demonstrate that ^18^F-NaF is a potentially useful biomarker, recent advances in imaging protocols have improved image quality and reproducibility of measurements, thereby enhancing this technique. A detailed description of standardized analysis techniques have been previously outlined.^16^,^17^ Utilization of contrast-enhanced computed tomography was an important advance, allowing accurate co-registration of the PET and CT scans by lining up the relatively high 18F-NaF activity in the blood pool compared to the myocardium with the left ventricular cavity visualized on contrast-enhanced CT in three orthogonal planes (Fig. 3). Moreover, this provides increased anatomical detail of the valve, allowing differentiating of ^18^F-NaF uptake in the valve from adjacent structures such as the aorta and coronary arteries. Motion correction techniques have proved similarly important. Motion of the valve throughout the cardiac cycle produces a wider distribution of PET signal, therefore, ECG-gating that captured the valve in diastole (50-75% RR interval) was employed to improve reproducibility.^16^,^18^ However, ECG-gating in this manner excludes 75% of potential data capture, increasing noise. Researchers have therefore developed a ‘motion correction’ algorithm that tracks the PET signal throughout each ECG gate and then collates all data to a single gate. This corrects for cardiac motion but does not involve data loss and therefore improves the signal-to-noise ratio (Fig. 4).^19^ Further iterations of this approach now allow additional correction for respiratory motion as well as bulk motion artifact.^20^ The final important advance has been in the approach to quantification. The ‘most diseased segment’ method, which averages the mean or maximum PET uptake values of the hottest two slices, precludes reproducibility issues associated with identifying the top and bottom slices of the valve.

When the above advances are combined, scan–rescan measurement error falls from >60% to 10%.^16^,^18^ With this degree of reproducibility, ^18^F-NaF becomes a crucial tool for elucidating novel insights in to the pathology of aortic stenosis and bioprosthetic valve degeneration as well as an efficacy endpoint in randomized controlled trials of novel therapies.

## ^18^F-Sodium Fluoride in Clinical Research

Using PET/CT ^18^F-sodium fluoride activity, the relationship between possible disease instigators and aortic valve disease can be investigated. Both lipoprotein(a) and oxidized phospholipids have been genetically linked to an increased incidence of aortic stenosis in the general population; however, whether these lipid markers are also associated with the propagation phase of aortic stenosis and faster disease progression remained uncertain.^21^-^23^ In a recent study of patients with calcific aortic valve disease, high levels of lipoprotein(a) or oxidized lipoprotein (the top tertile) were shown to be associated with higher baseline aortic valve ^18^F-NaF uptake, with this increased PET activity translating in to faster progression of the aortic valve CT calcium score, and more rapid progression of haemodynamic gradients on echocardiography.^24^ Raised oxidized phospholipids and lipoprotein(a) levels are therefore associated with an increased incidence and rate of progression in aortic stenosis and are therefore important potential therapeutic targets for patients with increased levels.^24^ Indeed, currently two randomized controlled trials are ongoing which assess the effect of niacin (NCT02109614) or PCSK9 inhibitors (NCT03051360) on lipoprotein(a) lowering and aortic stenosis progression; the latter using ^18^F-NaF uptake as a primary outcome.^22^

## ^18^F-Sodium Fluoride as a Clinical Tool in Native Aortic Valve Stenosis

Given the ability of ^18^F-NaF to measure disease activity and predict disease progression and clinical events in aortic stenosis, is there a role for this technique in clinical practice?^25^ The substantial costs and radiation exposure associated with ^18^F-NaF PET/CT means that it would need to provide incremental information to established echocardiographic and CT approaches. However, the studies reported to date have suggested that CT calcium scoring provides similar prognostic and predictive information to PET.^15^ In large part, this is due to the reasonably close association between baseline ^18^F-NaF PET activity and CT calcium scores. While at present this argues for wider use of CT and against routine ^18^F-NaF PET imaging, it does provide important pathophysiological insights. In particular, it suggests that calcium in the valve leaflets encourages further calcification activity and faster disease progression, perhaps via the increase in associated mechanical stresses within the valve. The result is a vicious cycle of progressive calcification that drives the propagation phase of aortic stenosis and suggests that calcification should be the predominant target for future therapies. Two ongoing randomized controlled trials are investigating this hypothesis, assessing the effect of denosumab or alendronate (SALTIRE II, NCT02132026) and vitamin K2 (BASIK 2, NCT02917525) on aortic stenosis progression (Table 1).^26^^18^F-NaF is being used as an efficacy endpoint in both trials.

**Table 1:** Ongoing clinical trials in aortic stenosis using ^18^F-NaF as an endpoint

Reference	Trial number	Design	Outcome measures
PCSK9 Inhibitors in the Progression of Aortic Stenosis	NCT03051360	140 patients mild-moderate aortic stenosis: 70 PCSK9 inhibitors vs 70 placebo	Primary: Change in aortic valve CT calcium score and Change in aortic valve ^18^F-NaF PET/CT
SALTIRE II	NCT02132026	150 patients with aortic *V*_max_ > 2.5 m/s and at least mild calcification seen on echo: 50 alendronic acid (70 mg/week) vs 50 Denosumab injection 6 monthly vs 50 placebo (25 tabs, 25 injection)	Primary: Change in valve calcium score Secondary: Change in valve ^18^F-NaF PET/CT
BASIK2	NCT02917525	44 patients with bicuspid aortic valve mild-moderate calcific aortic stenosis: 22 vitamin K2 (360 µg/day for 18 months) 22 placebo (for 18 months)	Primary: Change in ^18^F-NaF PET/MR

Further prospective studies are now ongoing to assess whether ^18^F-NaF PET imaging using the latest protocols and image analysis approaches can provide the incremental predictive information that would support a more extensive clinical role.

## Bioprosthetic Aortic Valve Degeneration

Bioprosthetic aortic valves are preferred in patients >65 years old because of reduced thrombogenicity and no requirement for long term anticoagulation. While there has been significant improvement in longevity since the introduction of bioprosthetic valves in the 1960s, the propensity towards structural deterioration over 10-20 years remains a major limitation to their use in younger patients.^27^,^28^ Typically, bioprosthetic valves are made of a covered frame with valve leaflets fashioned from either explanted porcine aortic valve or bovine pericardium. The pathological pathways underpinning bioprosthetic valve degradation are not fully understood, but are thought to involve microthrombus, pannus formation and excess mineralization consequent to plasminogen/fibrinogen, myofibroblast and macrophage-driven responses, respectively, quite different from those affecting the native valve.^29^-^32^ Structural valve degeneration (SVD) is defined as intrinsic leaflet deterioration associated with eventual haemodynamic dysfunction, and is categorized into four major stages: stage 0, no evidence of SVD; stage 1, SVD without significant haemodynamic changes (no/mild stenosis or regurgitation); stage 2, SVD with moderate stenosis or regurgitation; stage 3, SVD with severe stenosis or regurgitation, with re-intervention considered once symptoms develop in patients with severe disease.^33^ Standard assessment involves TTE (or transesophageal echocardiograph if TTE windows are poor) at baseline, one month after implantation and then annually to assess for changes in haemodynamic gradient.^4^,^33^,^34^ TTE/TEE is ideal for assessing haemodynamic changes and can identify gross leaflet abnormalities such as fluttering, thickening, or abnormal opening; however, echocardiography frequently only identifies end-stage valve degeneration with patients presenting in extremis and misses the earlier stage of its development. Computed tomography has been utilized to identify early and subtle changes on the valve which may not be seen on TTE/TEE, particularly thrombus and pannus formation, with hypoattenuated leaftlet thickening (HALT) seen in 4-7% of surgical bioprosthetic aortic valves at various periods after implantation.^35^,^36^ However, this imaging technique is also limited by imaging artifact related to the stent frame and motion in patients that frequently cannot receive beta-blockade. Moreover although CT can identify these changes, the clinical implications are not clear, with no difference in hemodynamic gradient or outcomes at one year in those with and without HALT.^37^ A biomarker which could sensitively detect the early stages of bioprosthetic degeneration and predict eventual deterioration would, therefore, fill an important area of clinical need.

**Figure 5:**
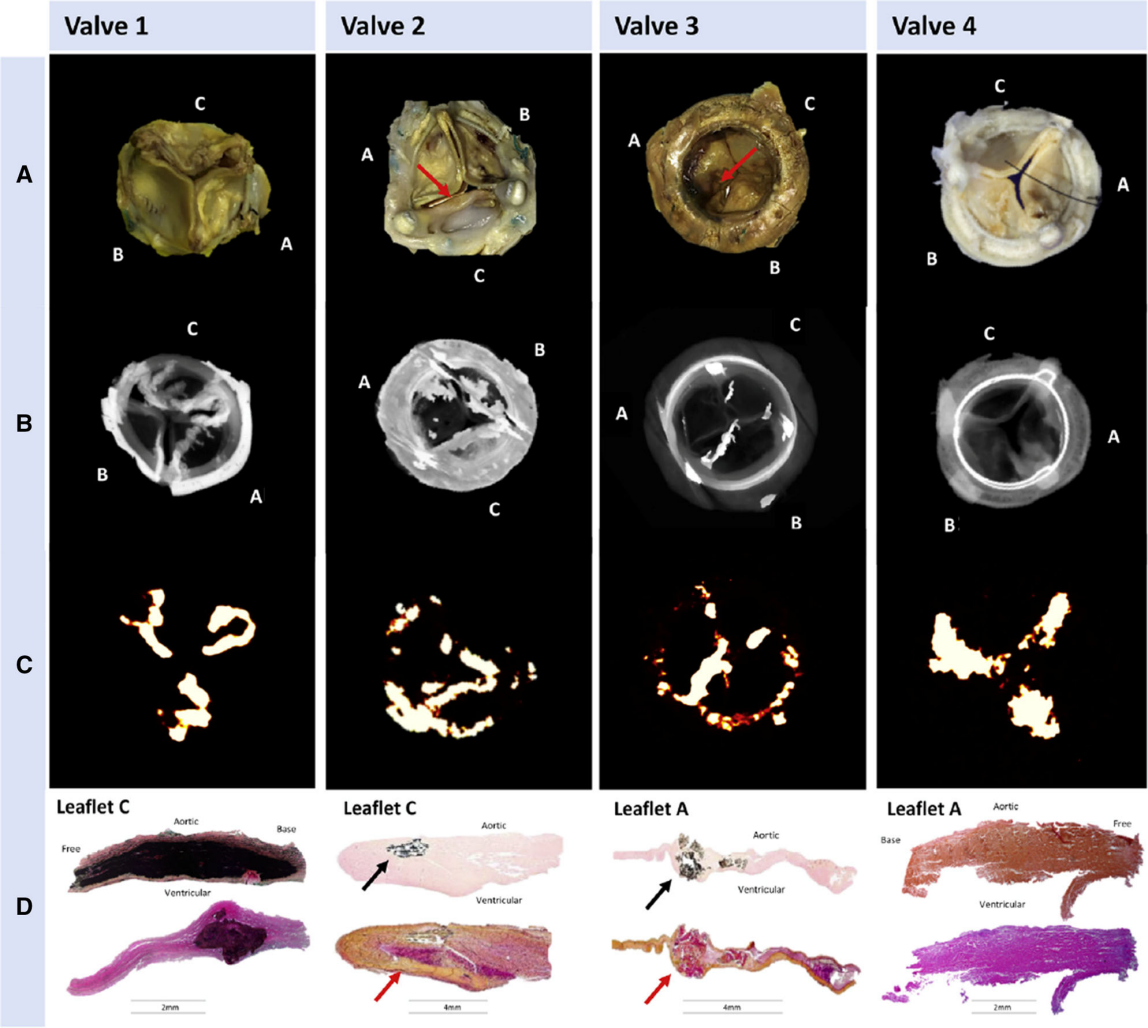
(Row **A**) Macroscopic visual appearances of failed and explanted bioprosthetic valves. (Row **B**) CT en face images of the valves. (Row **C**) PET en face images demonstrating increased ^18^F-fluoride uptake in all valves. (Row **D**) Histology staining of sections taken from valve leaflet as indicated, with von Kossa (top row, calcium appears black), Movat Pentachrome (bottom row, valves 1 and 4), and hematoxylin and eosin (bottom row, valves 2 and 3) stains. All 4 degenerate bioprostheses demonstrate increased ^18^F-fluoride uptake in the valve leaflets. In valve 1, this uptake corresponds to gross leaflet calcification observed macroscopically and on CT images with confirmation on histology (extensive black staining). In valve 2, increased ^18^F-fluoride uptake is observed in association with fibrotic leaflet thickening and pannus (red arrows) with associated calcification (black arrows) observed macroscopically and on CT with confirmation on histology. In valve 3, increased ^18^F-fluoride uptake is observed at the site of valve leaflet thrombus (red arrow) observed macroscopically at the base of leaflet 1, with confirmation of thrombus (red arrow) and colocalized calcification (black arrow) on histology. In valve 4, extensive ^18^F-fluoride uptake is observed in the absence of calcification on CT and histology but instead in areas of leaflet thickening, marked fluid insudation, and disrupted collagen architecture. *CT*, computed tomography; *PET*, positron emission tomography. Adapted from Timothy et al.^38^ Under creative commons licence

**Figure 6:**
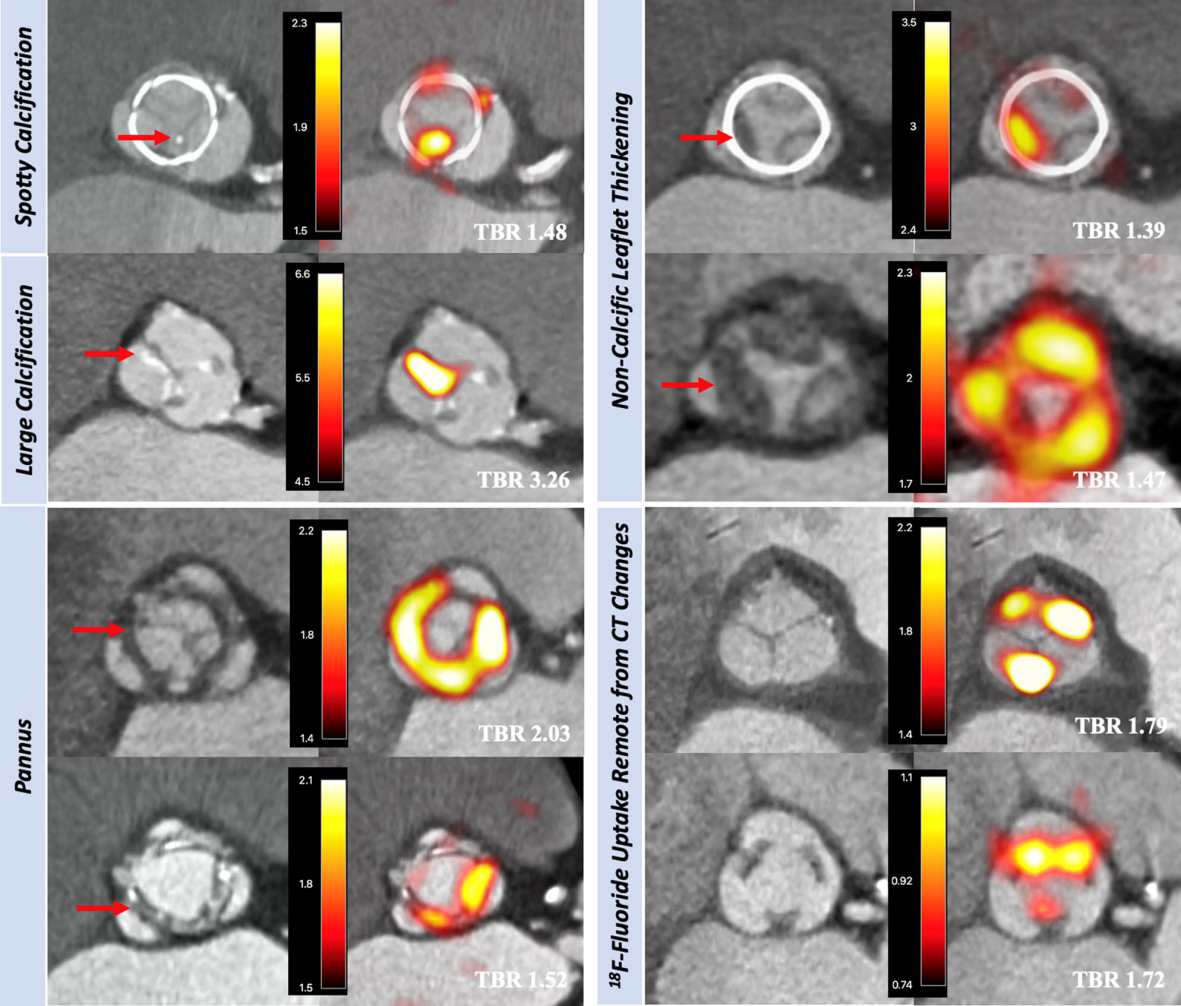
Baseline CT (left) and ^18^F-fluoride PET (right) images from patients with bioprosthetic aortic valves. En face CT images of aortic bioprosthetic valves showing spotty calcification and large calcification (top left), circumferential pannus (bottom left), and noncalcific leaflet thickening suggestive of thrombus (top right) (all abnormalities identified by red arrows). Hybrid en face PET-CT images in the same patients: increased bioprosthetic ^18^F-fluoride activity (red/yellow areas) is observed in each patient colocalizing with the CT abnormalities. ^18^F-fluoride activity was also commonly observed remote from leaflet changes on CT (bottom right). Scale bars in the center of each pair of images represent standardized uptake values (SUV). Target-to-background (TBR) values are annotated on the hybrid PET-CT images in white text. Adapted from Timothy et al.^38^ Under creative commons licence

**Figure 7:**
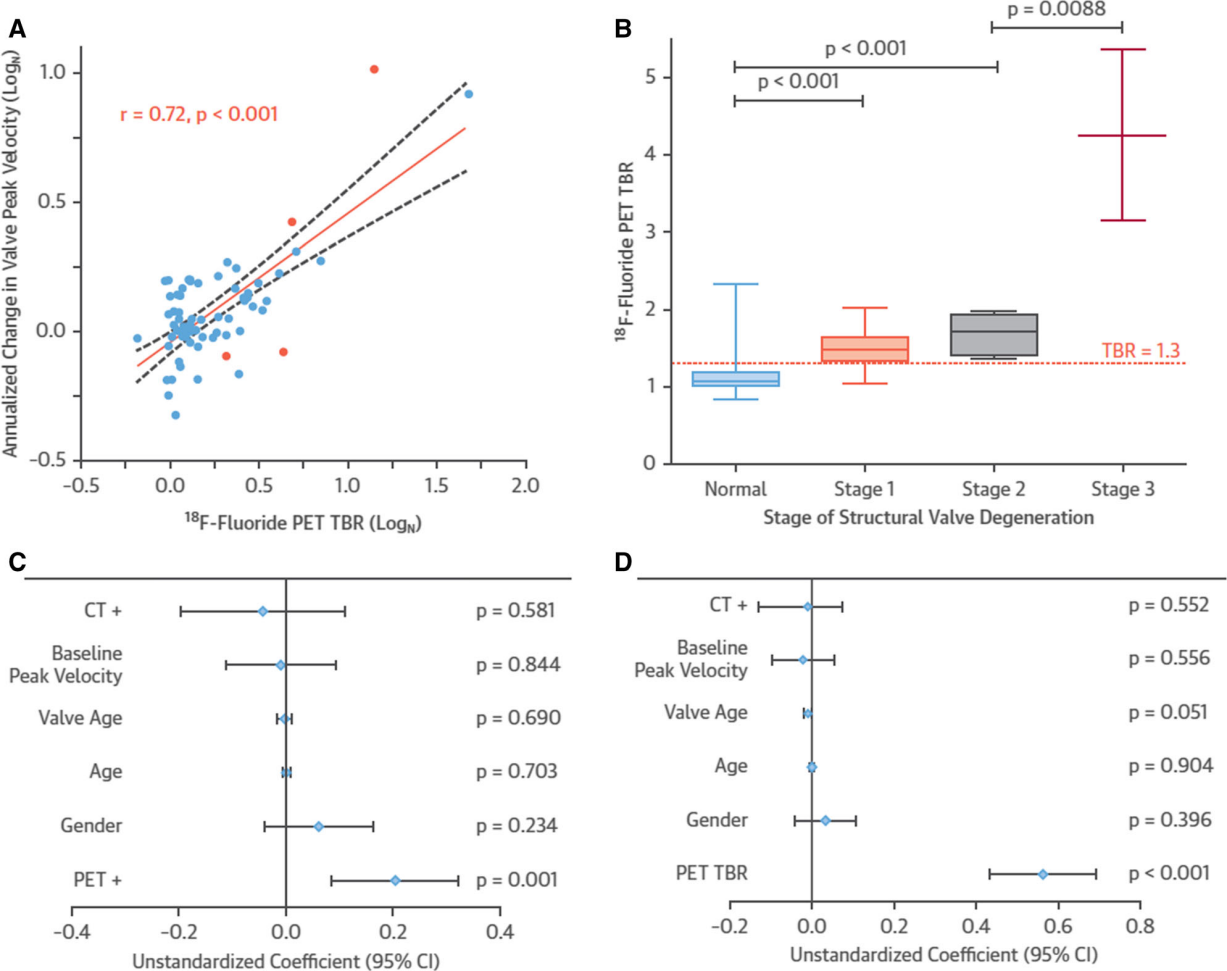
(**A**) A strong correlation was observed between baseline ^18^F-fluoride uptake in the bioprosthetic valves (TBR) and subsequent progression in bioprosthetic valve peak velocity (log transformation applied; *r* = 0.72; *P* < .001). Orange dots signify patients who developed new bioprosthetic valve regurgitation during follow-up. (**B**) ^18^F-fluoride uptake (dashed orange line represents threshold for increased ^18^F-fluoride uptake; TBR 1.3) in patients with different stages of structural valve degeneration after 2-year follow-up (stage 0: no significant change from post-implantation [n = 54]; stage 1: morphological abnormalities without significant hemodynamic changes [n = 9]; stage 2: new moderate stenosis and/or regurgitation [n = 5]; stage 3: new severe stenosis and/or severe regurgitation [n = 2]) demonstrating incrementally higher uptake values with increasing severity of structural valve degeneration. (**C** and **D**) Forest plots of unstandardized coefficients (95% confidence intervals) from a multivariable linear regression analysis predicting change in bioprosthetic valve function (annualized change in peak velocity) during follow-up. When examining all relevant baseline characteristics, ^18^F-fluoride uptake was the only independent predictor of hemodynamic deterioration in valve function when used both as a dichotomous variable (PET, TBR > 1.3) (**C**) and as a continuous variable (TBR) (**D**). *CI*, confidence interval. Adapted from Timothy et al.^38^ Under creative commons licence

As bioprosthetic valve leaflet calcification appears central to structural deterioration, researchers investigated the relationship between SVD and calcification *ex vivo* using explanted surgical bioprosthetic valves and ^18^F-NaF microPET/CT. Various pathological processes, including overt nodular calcification, thrombus, pannus and non-specific leaflet thickening were all visually highlighted by ^18^F-NaF, and correlated with pathology on histological staining (Fig. 5), suggesting calcification as a common endpoint for a variety of different triggers to bioprosthetic degeneration.^38^ In a prospective study, 80 patients recruited 1 month - 20 years after surgical bioprosthetic aortic valve replacement received baseline ^18^F-NaF PET/CT and TTE, with follow-up at 2 years. The same pathological processes were identified *in vivo* as seen in the explanted tissue (Fig. 6).^38^ Seventy one patients who had no echocardiographic evidence of valve dysfunction at baseline, 19% were found have pathological CT change, while a third had evidence of increased ^18^F-sodium fluoride uptake. Of the 67 who had available follow-up data, 10 (15%) developed new valve dysfunction, with two requiring urgent valve replacement and one death directly related to valve failure. Crucially, in multivariable linear regression analysis, baseline ^18^F-NaF was the only predictor of bioprosthetic valve dysfunction outperforming age, echocardiographic findings, CT findings, valve age and gender (Fig. 7). The patients who developed overt valve failure all had intense ^18^F-NaF baseline activity. ^18^F-NaF PET/CT therefore holds major promise for the early detection of bioprosthetic valve degeneration with potentially important clinical implications on the intensity of follow-up, and timing of replacement surgery for patients. Establishing the clinical role for ^18^F-NaF PET/CT in these patient groups will require validation in larger multicenter prospective studies.

## Conclusions

Aortic stenosis and bioprosthetic aortic valve degeneration represent major health problems that, despite considerable research effort, currently lack effective preventative medical therapies. Calcification activity, as quantified non-invasively using ^18^F-NaF PET/CT, has been identified as the primary driver of both types of disease. Recent optimization of ^18^F-NaF PET has made it a sensitive and reproducible marker of aortic stenosis disease activity, providing important pathological insights and an efficacy endpoint in multiple ongoing randomized controlled trials. However, perhaps the most promising clinical translation for ^18^F-NaF PET lies in bioprosthetic aortic valve degeneration, where it can provide an early assessment of valve degeneration not currently offered by other imaging techniques.

## Electronic supplementary material

Below is the link to the electronic supplementary material.

Electronic supplementary material 2 (M4A 5058 kb)

Electronic supplementary material 1 (PPTX 1851 kb)

## Abbreviations

^18^F-NaF: ^18^F-sodium fluoride
HALT: Hypoattenuated leaflet thickening
PET: Positron emission tomography
CT: Computed tomography
TAVI: Transcatheter aortic valve implantation
TTE: Transthoracic echocardiography
TEE: Transesophageal echocardiography
