# Supplementary material for: Detecting native and bioprosthetic aortic valve disease using 18F-sodium fluoride: Clinical implications
Source: J Nucl Cardiol. 2020 Nov 11;28(2):481–91. doi: 10.1007/s12350-020-02411-x (PMC8076133; doi:10.1007/s12350-020-02411-x)
Supplement: Supplementary file 2 — Electronic supplementary material 1 (PPTX 1851 kb) [file 12350_2020_2411_MOESM2_ESM.pptx]

## Slide 1
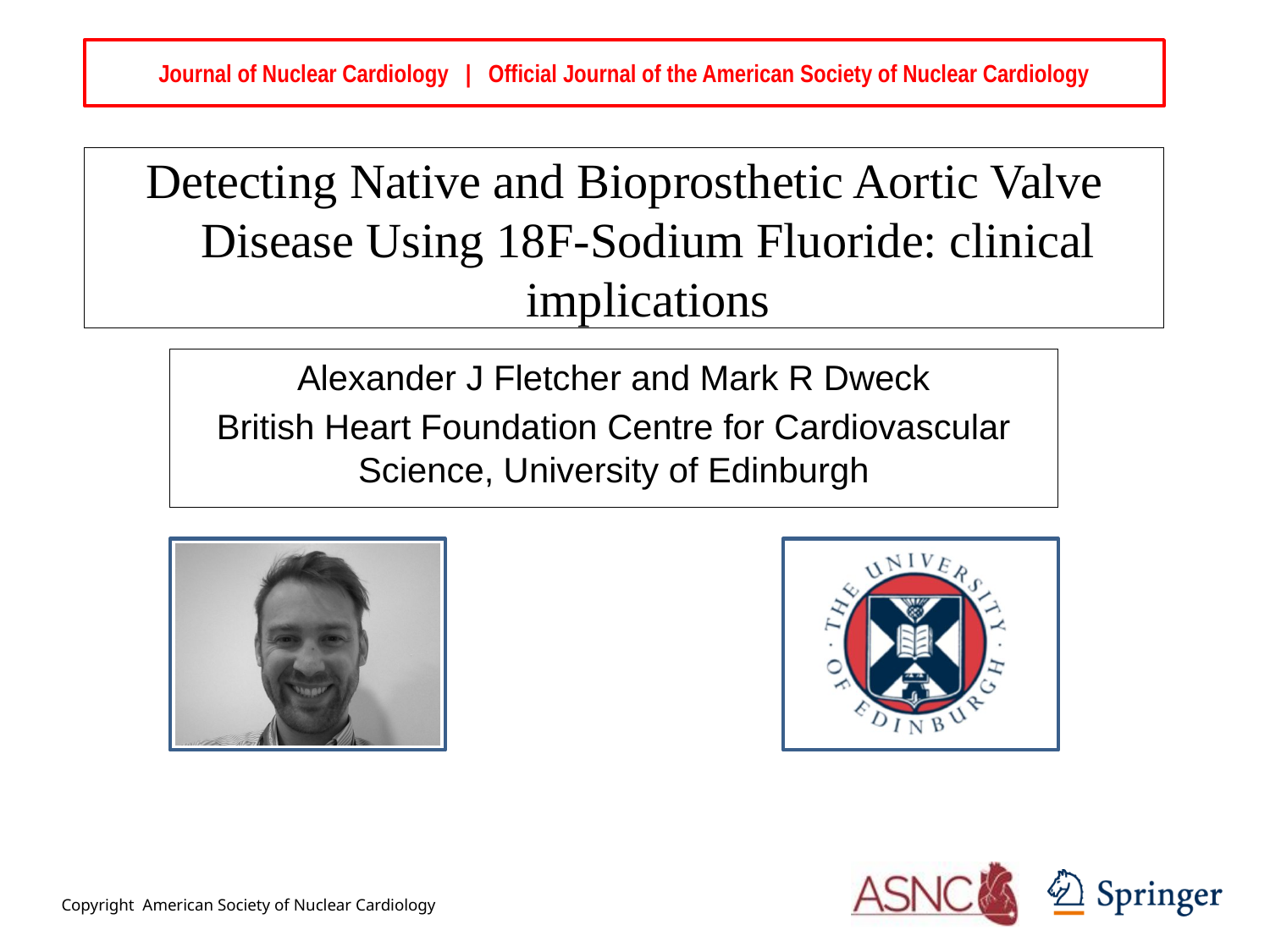

Journal of Nuclear Cardiology | Official Journal of the American Society of Nuclear Cardiology
# Detecting Native and Bioprosthetic Aortic Valve Disease Using 18F-Sodium Fluoride: clinical implications
Alexander J Fletcher and Mark R Dweck
British Heart Foundation Centre for Cardiovascular Science, University of Edinburgh
Head shot of author
required
Institution
Picture/Logo
Optional
Copyright American Society of Nuclear Cardiology

## Slide 2
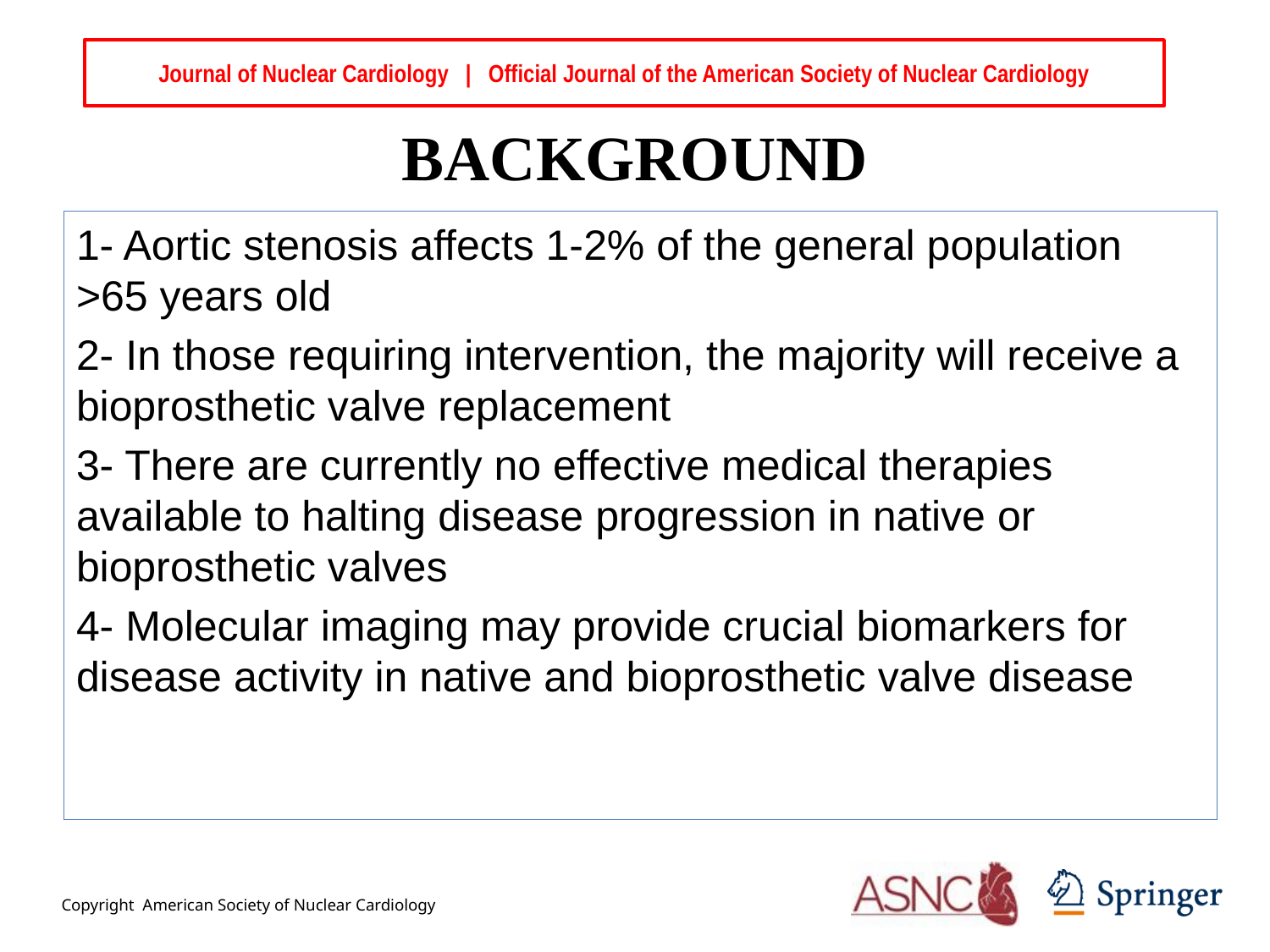

Journal of Nuclear Cardiology | Official Journal of the American Society of Nuclear Cardiology
# BACKGROUND
1- Aortic stenosis affects 1-2% of the general population >65 years old
2- In those requiring intervention, the majority will receive a bioprosthetic valve replacement
3- There are currently no effective medical therapies available to halting disease progression in native or bioprosthetic valves
4- Molecular imaging may provide crucial biomarkers for disease activity in native and bioprosthetic valve disease
Copyright American Society of Nuclear Cardiology

## Slide 3
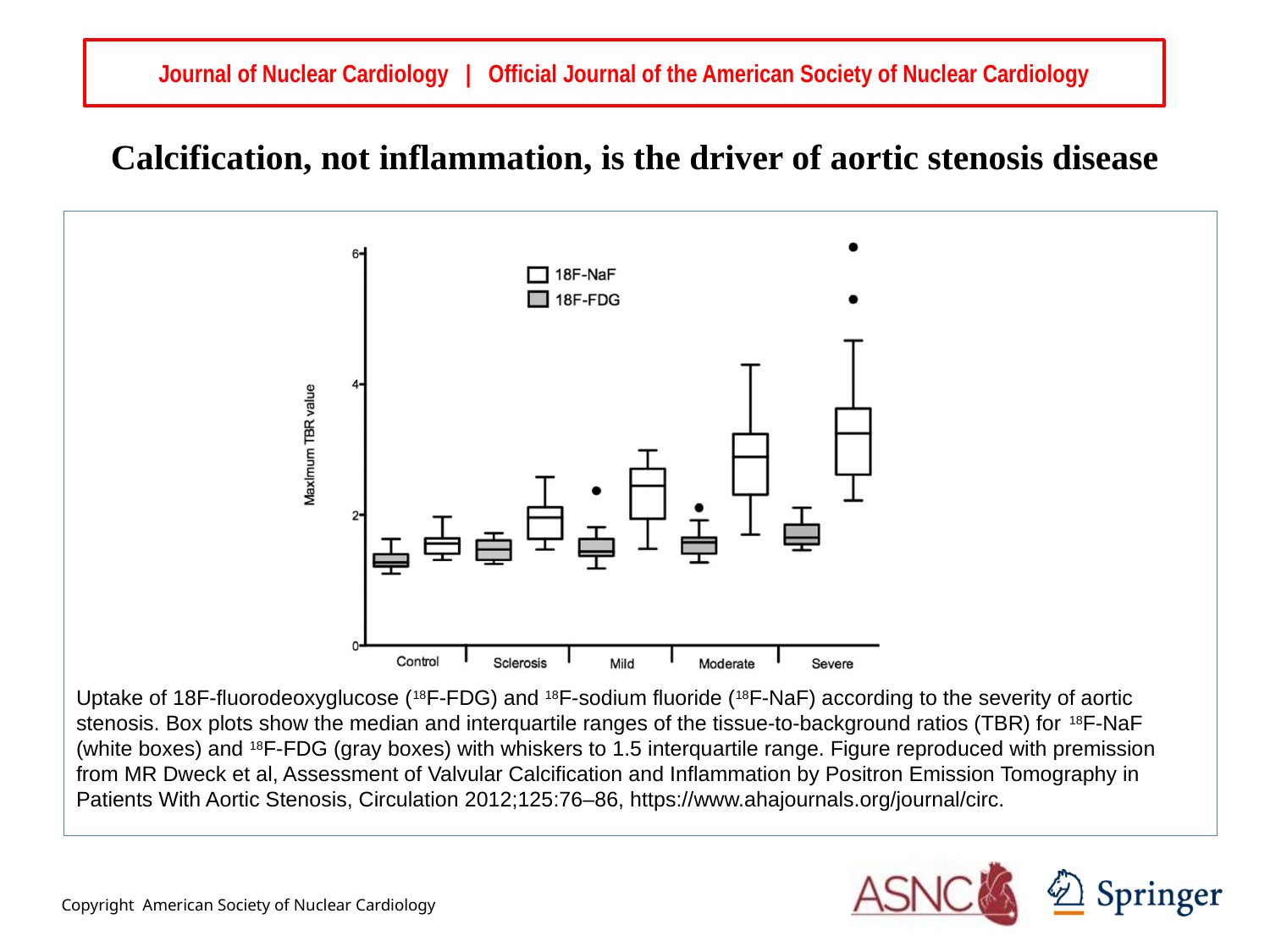

Journal of Nuclear Cardiology | Official Journal of the American Society of Nuclear Cardiology
# Calcification, not inflammation, is the driver of aortic stenosis disease
Uptake of 18F-fluorodeoxyglucose (18F-FDG) and 18F-sodium fluoride (18F-NaF) according to the severity of aortic stenosis. Box plots show the median and interquartile ranges of the tissue-to-background ratios (TBR) for 18F-NaF (white boxes) and 18F-FDG (gray boxes) with whiskers to 1.5 interquartile range. Figure reproduced with premission from MR Dweck et al, Assessment of Valvular Calcification and Inflammation by Positron Emission Tomography in Patients With Aortic Stenosis, Circulation 2012;125:76–86, https://www.ahajournals.org/journal/circ.
Copyright American Society of Nuclear Cardiology

## Slide 4
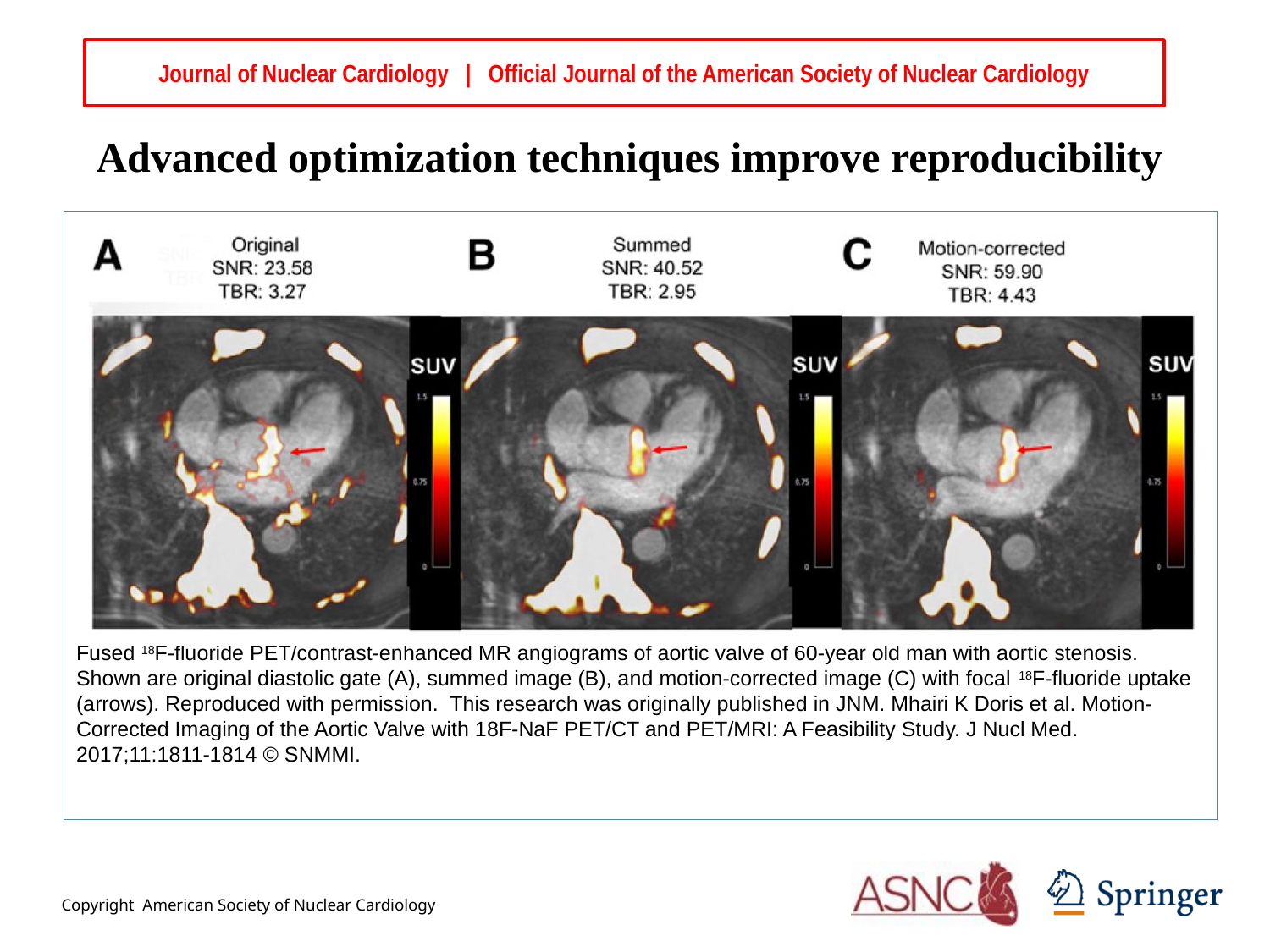

Journal of Nuclear Cardiology | Official Journal of the American Society of Nuclear Cardiology
# Advanced optimization techniques improve reproducibility
Fused 18F-fluoride PET/contrast-enhanced MR angiograms of aortic valve of 60-year old man with aortic stenosis. Shown are original diastolic gate (A), summed image (B), and motion-corrected image (C) with focal 18F-fluoride uptake (arrows). Reproduced with permission. This research was originally published in JNM. Mhairi K Doris et al. Motion-Corrected Imaging of the Aortic Valve with 18F-NaF PET/CT and PET/MRI: A Feasibility Study. J Nucl Med. 2017;11:1811-1814 © SNMMI.
Copyright American Society of Nuclear Cardiology

## Slide 5
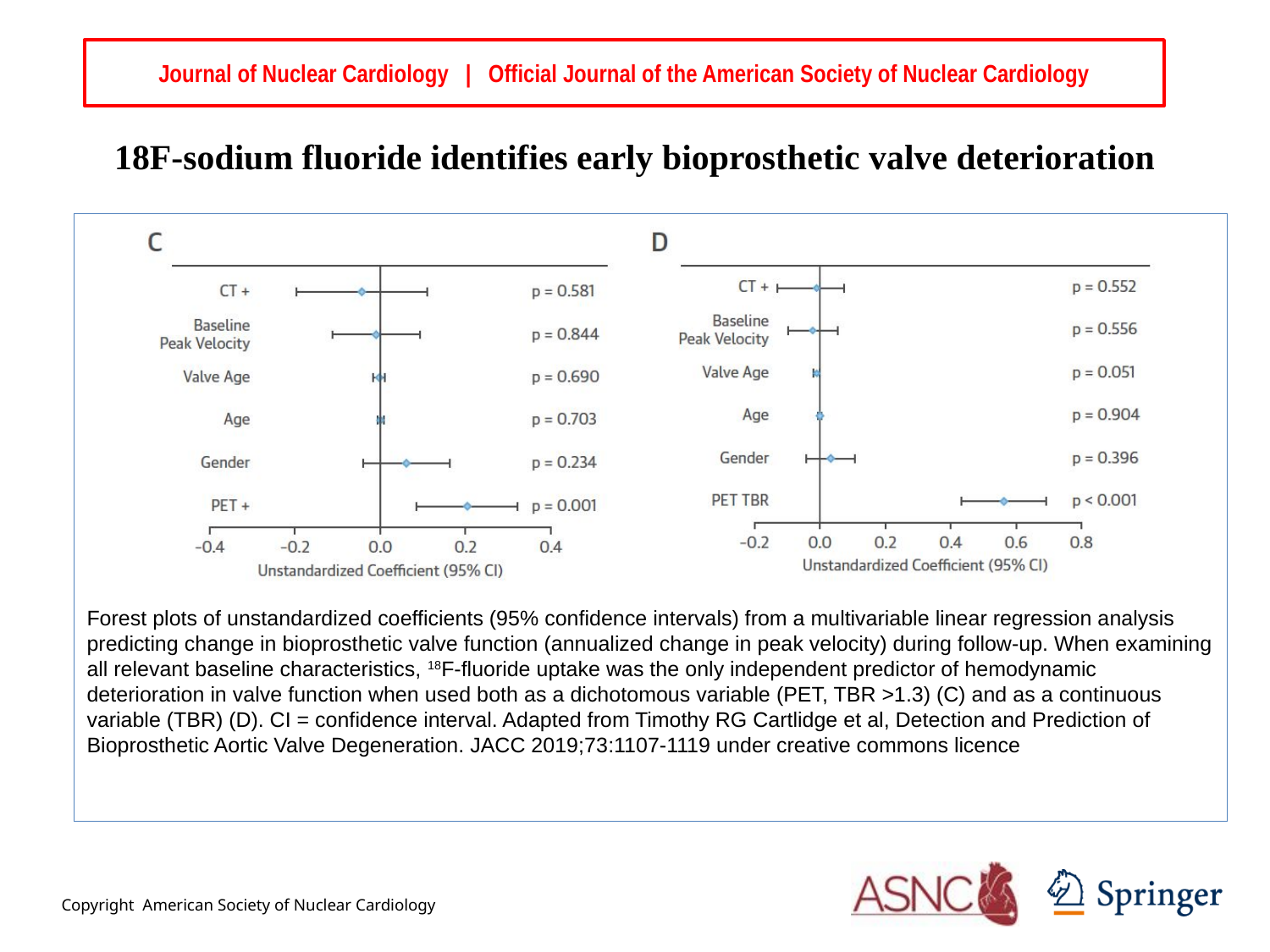

Journal of Nuclear Cardiology | Official Journal of the American Society of Nuclear Cardiology
# 18F-sodium fluoride identifies early bioprosthetic valve deterioration
Forest plots of unstandardized coefficients (95% confidence intervals) from a multivariable linear regression analysis predicting change in bioprosthetic valve function (annualized change in peak velocity) during follow-up. When examining all relevant baseline characteristics, 18F-fluoride uptake was the only independent predictor of hemodynamic deterioration in valve function when used both as a dichotomous variable (PET, TBR >1.3) (C) and as a continuous variable (TBR) (D). CI = confidence interval. Adapted from Timothy RG Cartlidge et al, Detection and Prediction of Bioprosthetic Aortic Valve Degeneration. JACC 2019;73:1107-1119 under creative commons licence
Copyright American Society of Nuclear Cardiology

## Slide 6
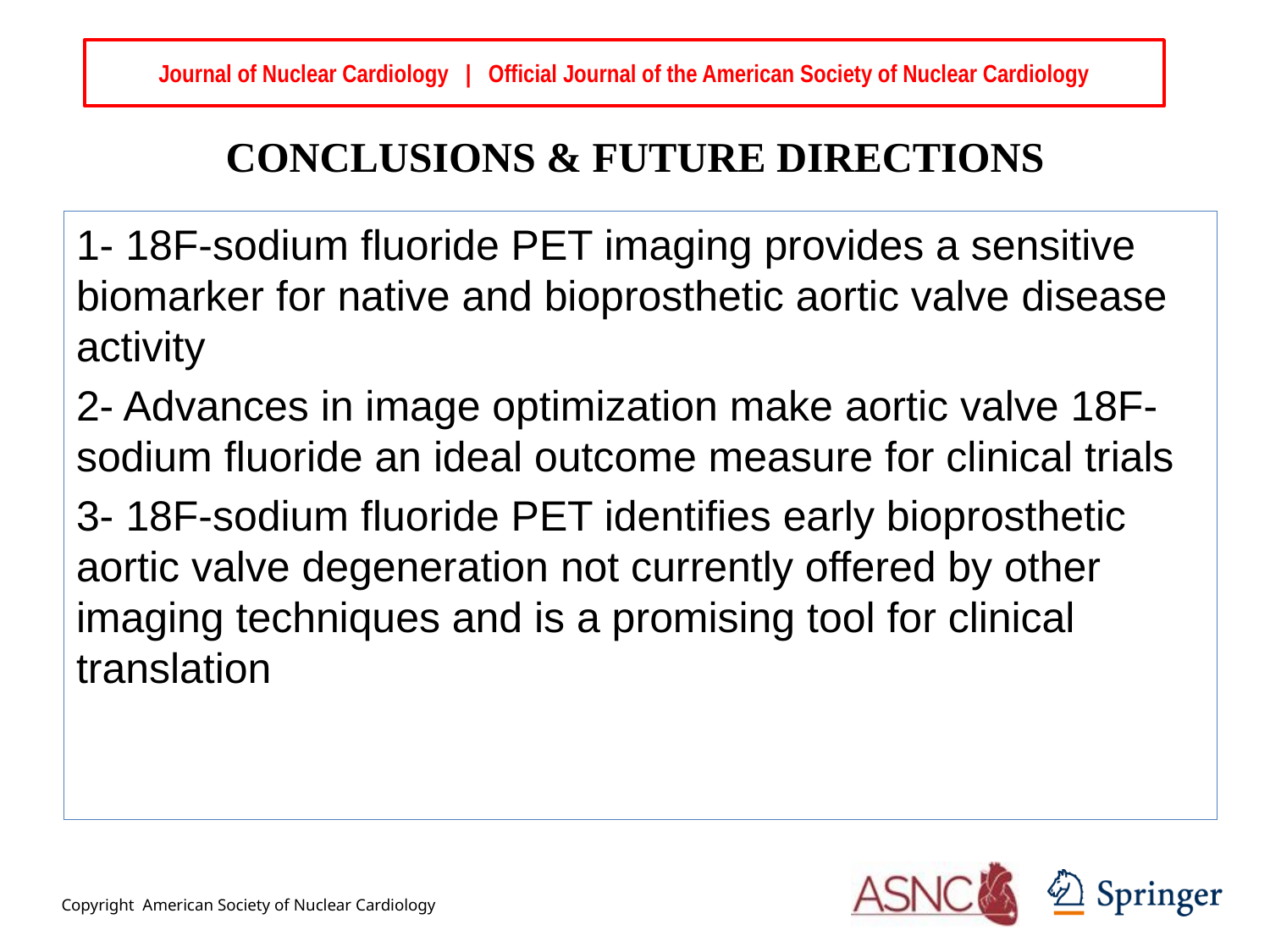

Journal of Nuclear Cardiology | Official Journal of the American Society of Nuclear Cardiology
# CONCLUSIONS & FUTURE DIRECTIONS
1- 18F-sodium fluoride PET imaging provides a sensitive biomarker for native and bioprosthetic aortic valve disease activity
2- Advances in image optimization make aortic valve 18F-sodium fluoride an ideal outcome measure for clinical trials
3- 18F-sodium fluoride PET identifies early bioprosthetic aortic valve degeneration not currently offered by other imaging techniques and is a promising tool for clinical translation
Copyright American Society of Nuclear Cardiology
